# Supplementary material for: Fractionation-Dependent Radiosensitization by Molecular Targeting of Nek1
Source: Cells. 2020 May 16;9(5):1235. doi: 10.3390/cells9051235 (PMC7291120; doi:10.3390/cells9051235)
Supplement: Supplementary file 1 [file cells-09-01235-s001.pdf]

Supplemental Figures

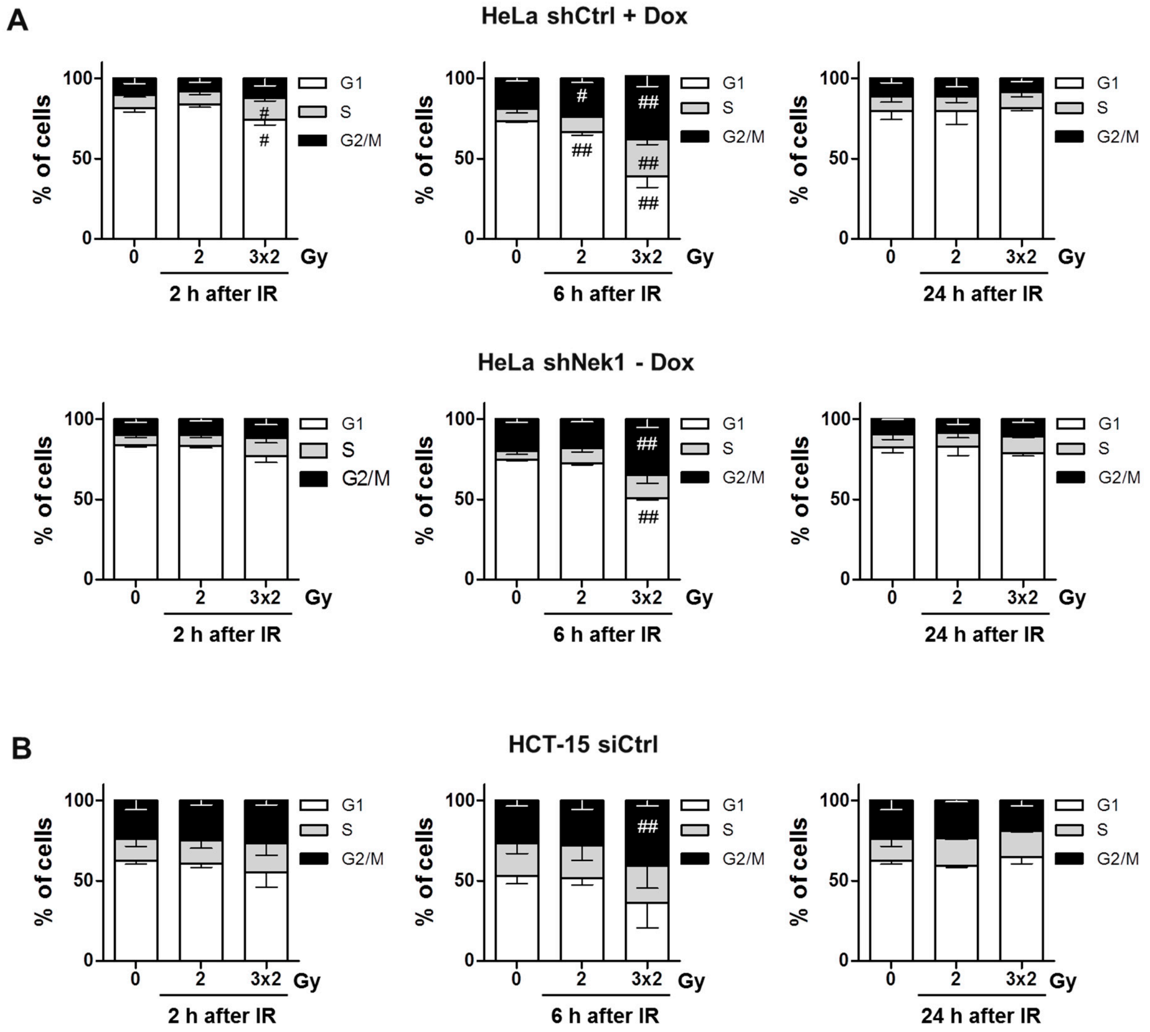

Figure S1. (**A**) Cell cycle distribution of HeLa shCtrl cells treated with Dox and Hela shNek1 in the absence of Dox or (**B**) HCT-15 cells transfected with siCtrl, irradiated with either a single dose of 2 Gy, or fractionated 3 x 2 Gy with an interval of 2 h (left), 6 h (middle) and 24 h (right) analyzed by flow cytometry (n = 3; # $p < 0.05$, ## $p < 0.01$ irradiated vs. non irradiated control).

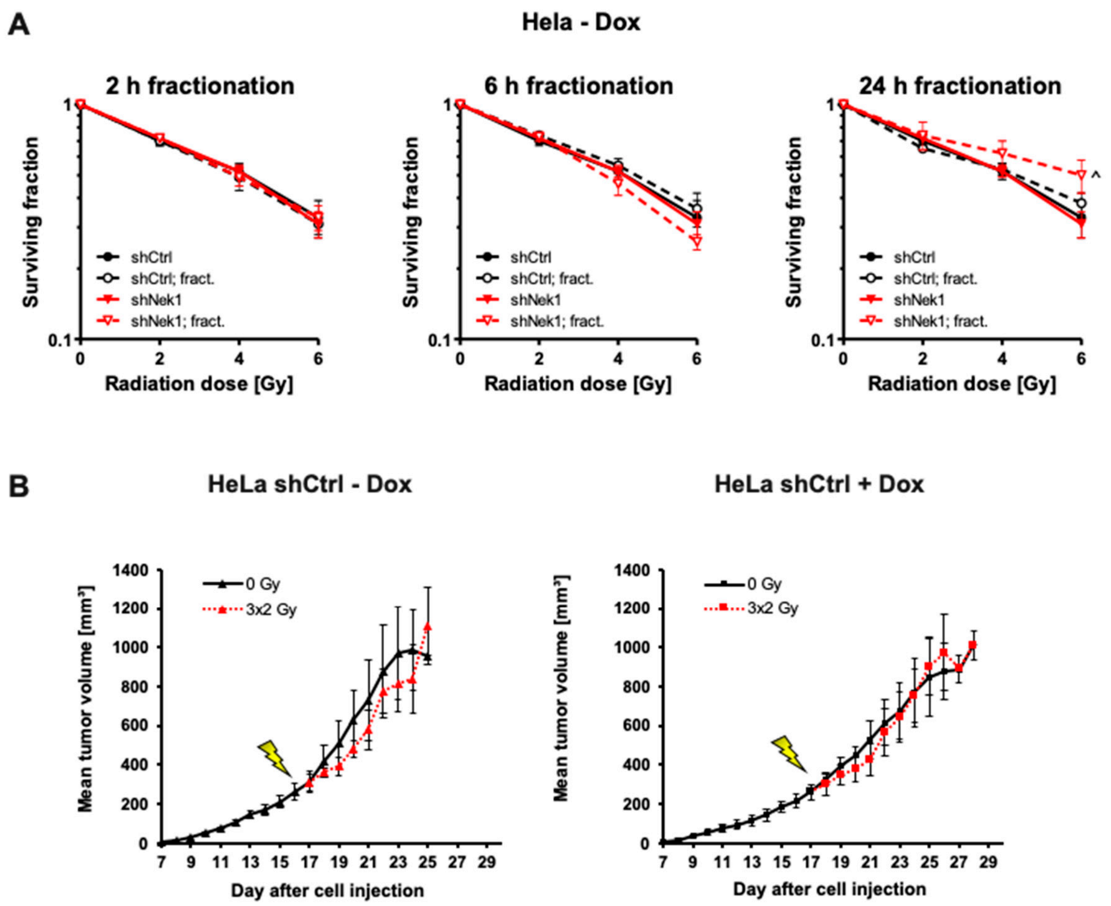

Figure S2. (**A**) 3D clonogenic radiation survival following a 2, 4 or 6 Gy single dose irradiation or fractionated irradiation with 2 h (left), 6 h (middle) and 24 h fractionation interval (right) of HeLa shNek1 and shCtrl cells in the absence of Dox (means ± SD; n = 3; ^ $p < 0.05$ fractionated vs. single dose). (**B**) Relative tumor growth curves of NSG mice (6 animals per group) inoculated with HeLa shCtrl cells treated with (right) or without Dox (left). Mean values of tumor volumes for each treatment group are shown.

Supplemental Table 1

Table S1: Correlation of Nek1 expression with clinicopathologic characteristics. Abbreviation: FIGO: Fédération Internationale de Gynécologie et d'Obstétrique.

	No. of patients	Nek1 WS ≤ 6 n (%)	Nek1 WS > 6 n (%)	p-value
**Age**				
≤ 59 years	38	22 (29.7)	16 (21.6)	
> 59 years	36	21 (28.3)	15 (20.2)	0.970
**T-Stage**				
T1/2	39	22 (29.7)	17 (22.9)	
T3/4	35	24 (32.4)	11 (14.8)	0.288
**N-Stage**				
N0	39	25 (33.7)	14 (18.9)	
N1	34	21 (29.3)	13 (16.5)	
Nx	1		1 (1.3)	0.652
**M-Status**				
M0	63	40 (54.0)	23 (31.0)	
M1	11	6 (8.1)	5 (6.9)	0.579
**FIGO**				
Low (Ia-IIb)	29	18 (24.3)	11 (14.8)	
High (IIIa-IVb)	45	28 (37.8)	17 (22.9)	0.99
**Grading**				
G1/2	38	21 (29.3)	17 (22.9)	
G3	34	23 (31.0)	11 (14.8)	
Gx	2	2 (2.70)		0.171
**p16^{INK4a}**				
Low (WS ≤ 6)	27	17 (22.9)	10 (13.5)	
High (WS > 6)	47	29 (39.2)	18 (24.3)	0.916
